# Proximal femur anatomy-implant geometry discrepancies

**DOI:** 10.1051/sicotj/2022004

**Published:** 2022-03-04

**Authors:** Andries Johannes Cornelissen, Nando Ferreira, Marilize Cornelle Burger, Jacobus Daniel Jordaan

**Affiliations:** Division of Orthopaedic Surgery, Department of Surgical Sciences, Faculty of Medicine and Health Sciences, Stellenbosch University Cape Town 7505 South Africa

**Keywords:** Femur Neck-Shaft axis offset, Implant mismatch, Femur Geometry, Cephalomedullary Nails

## Abstract

*Objectives*: Due to ongoing concern about femur anatomy-implant mismatches, this cross-sectional study aimed to create a geometric femur profile and used it to identify and quantify possible mismatches between femur anatomy and cephalomedullary nail dimensions. The work further aimed to assess whether patient demographics affect anatomy-implant coherence. *Methods*: One hundred skeletally mature complete femur computer tomography (CT) scans were collected and exported to software enabling landmark placement and measures with multiplanar reconstruction techniques. *Results*: Clinically relevant anatomy-implant discrepancies included the femur neck and shaft axis offset 6.1 ± 1.7 mm (95% CI [5.7–6.4]), femur radius of curvature 1.2 ± 0.3 m (95% CI [1.1–1.2]), femur anteversion 18.8 ± 9.2 (95% CI [16.9–20.6]). The implants reviewed in this study did not compensate for the femur neck and shaft axis offset and had a larger radius of curvature than the studied population. Clinically significant demographic geometry differences were not identified. *Conclusion*: There were discrepancies between femur anatomy and cephalomedullary nail implant design; however, no clinically significant femur feature inconsistency was identified among the demographic subgroups. Due to the identified anatomy-implant discrepancies, including the femur neck and shaft axis offset, we suggest that these measurements be considered for future implant design and surgical technique.

## Introduction

Proximal femur fracture incidence rises due to aging populations and increased trauma events [1, 2]. Reconstruction of proximal femur anatomy and accurate implant placement remains paramount to negate fixation failure [3, 4].

Contemporary cephalomedullary nail (CMN) designs rely on the concept of anatomy coherence and an intersecting femur shaft and neck axis for a secure fit between the nail and the cephalic lag screw [5, 6]. However, despite the multiple intraoperative implant dimension choices, various discrepancies between anatomy and modern implants remain a challenge to successful surgery [3]. Recent computer tomography (CT) three-dimensional image interpretation techniques have facilitated recognition and anticipation of complex anatomy-implant mismatches [7]. Such studies have generally compared measurement methods or investigated a single measurement rather than exploring the interplay of dimensions that impact fixation [3, 7].

Literature also suggests femoral anatomy and implant design mismatch are influenced by patient demographics, namely ethnicity, age, sex, and age [8–10]. Universal implants, however, are often employed worldwide across demographic groups without implant differentiation [10].

This study aimed to establish a planar corrected computed tomography geometric profile of the proximal femur, perform a demographic subgroup analysis, and compare the findings to contemporary implants utilized to manage fractures in this anatomical area. The result of this study intends to identify whether implants are compatible with anatomy parameters or need to be differentiated for demographic or geographic subgroups.

## Material and methods

This cross-sectional study assessed one hundred complete femur CT scans of adults who underwent imaging for medical indications at a high-volume tertiary hospital in South Africa between January 2020 and April 2020.

Inclusion required complete femur imaging with a slice thickness of less than one millimeter. Scans were excluded if there was an indication of previous or current trauma, neoplastic conditions, congenital deformity, image artifacts, incomplete radiologic data, or image depiction, which prompted collection over four months.

Scans were performed by a Siemens SOMATOM Definition Edge 128 (Siemens, Erlangen, Germany). The detector collimation was 5 × 128 × 0.6 mm, employing z-Sharp Technology. The settings for pitch, rotation speed, fixed tube current-time product, and tube voltage were 0.4, 0.5 s, 84 mAs, and 120 kVp, respectively. The Phillips IntelliSpace (Phillips, Amsterdam, Netherlands) Picture Archiving and Communication System (PACS) software was used to anonymize the data after recording the patient’s age, sex, and femur side.

The anonymized CT data was imported to Stratovan Checkpoint, 3D shapes, and morphometrics software [11]. The CT images were then orientated to the desired anatomical planes with multiplanar reconstruction techniques to allow landmark placement and measurements according to a standardized protocol (see Text, Supplemental Online Material 1. Text outlining the steps to obtain the measures). A single investigator performed the initial landmark placement, which an additional investigator reviewed. Finally, the angles and distances between the defined points were measured using tools within the software application, which did not involve subjective operator involvement.

Data were analyzed using Statistica version 13.5 (TIBCO software). Normally distributed variables are reported as means ± standard deviations with 95% confidence intervals (CI) where appropriate, while not normally distributed variables are reported as medians (interquartile ranges, IQR). Differences between groups (such as male/female or left/right) were investigated using two-tailed *t*-tests or Mann-Whitney U tests, while Pearson’s correlations or Spearman Rank correlations were used to examining relationships between variables parametric and non-parametric data, respectively.

## Results

A total of 155 CT scans were identified, 55 scans were excluded due to incomplete inclusion of the normal femur, image slices larger than 1 mm, image artifacts, or pathology involving the femur. One hundred met inclusion criteria and were used for anatomy dimension analysis. The final cohort of scans was well distributed with 56 male and 44 female patients and included 51 right and 49 left femurs. The mean age of participants was 35.2 years ± 13.5 (95% CI [32.6–37.9]), ranging from 18 to 75 years. Table 1 summarizes this study’s principal femur measurements, while Table 2 lists a comparative table of commercially available CMNs with the corresponding measures. The Gamma 3 Nail (Kalamazoo, Michigan, USA) and Smith and Nephew Intertan (Watford, UK) were used for comparison to the obtained femur dimensions [5, 6].

**Table 1:** Measurement data of the femur.

Measurement description	Mean ± *SD*	95% CI
Femur neck-shaft axis offset (mm)	6.1 ± 1.7	5.7–6.4
GT to sLSNI (mm)	40.3 ± 5.1	39.3–41.4
Femur neck axis length (mm)	94.1 ± 7.4	92.6– 95.6
NSA of proximal femur (°)	126.3 ± 5.4	125.2–127.4
NSA to apex of femur bow (°)	126.5 ± 5.6	125.4–127.6
NSA to DICN (°)	128.3 ± 5.6	127.2–129.4
Radius of curvature (m)	1.2 ± 0.3	1.1–1.2
Femoral anteversion (°)	18.8 ± 9.2	16.9–20.6

Abbreviations: *SD*, standard deviation; 95% CI, 95 percent confidence interval; mm, millimeter; NSA, neck-shaft angle; DICN, distal intercondylar notch; m, meter; GT, greater trochanter tip; sLSNI, shaft lag screw nail interface.

**Table 2:** Anatomical measurements from the present study compared to the cephalomedullary nail designs parameters of the Stryker Gamma 3 Nail (Kalamazoo, Michigan, USA) [6] and the Smith and Nephew Intertan (Watford, UK) [5].

Measurement description	Mean ± *SD*	Gamma 3^a^	Intertan^a^
Femur neck-shaft axis offset (mm)	6.1 ± 1.7		
GT to sLSNI (mm)	40.3 ± 5.1	38.4, 42, 46 (end caps to 10 mm)	35.6 (with 125° angled nail)
		The length depends on the length of the nail.	
Femur neck axis length (mm)	94.1 ± 7.4	70–130	70–125
NSA of proximal femur (°)	126.3 ± 5.4	120, 125, 130	125, 130
Radius of curvature (m)	1.2 ± 0.3	1.5, 2	1.5
Femur anteversion (°)	18.8 ± 9.2	10	12

Abbreviations: *SD*, standard deviation; mm, millimeter; NSA, neck-shaft angle; m, meter; GT, greater trochanter tip; sLSNI, shaft, and lag screw nail interface.

a Missing value are those that were not reported in the device brochures. The implant parameters are obtained from the product brochures [5, 6]

The femur neck axis persistently passed anterior to the proximal femur axis, and the offset (FNSAO) measured 6.1 mm ± 1.7 (95% CI [5.7–6.4]); however, there was no comparable offset within the design of CMNs. No correlation between age and FNSAO was observed (*r* = 0.116). Additionally, no correlations were observed between FNSAO and other measurements reported in this study (Supplemental Online Material 2. Table Listing Correlations Between Various Measures and the Femur Neck-Shaft Axis Offset). The length from the greater trochanter tip to the point of closest proximity between FNSAO measured 40.3 mm ± 5.1 (95% CI [39.3–41.4]), which is equivalent to the length offered by CMNs. Sampled CMN lag screw length ranged from 75 to 130 mm and encompassed the measured femur neck axis length of 94.1 mm ± 7.4 (95% CI 92.6– 95.6).

Femur neck-shaft angle (NSA) was measured with three definitions, of which the definition based on the proximal femur was compared with the obtuse angle between the CMN lag screw and nail. A proximal NSA of 126.3° ± 5.4 (95% CI [125.2–127.4]) was identified, which falls within the range of the CMNs (120–130°). However, the range of measured NSA (106.96–141.3°) fell outside of the range of the sampled CMNs.

Femur anteversion measured 18.8° ± 9.2 (95% CI [16.9–20.6]), which is larger than the CMN anteversion of 10 and 12° for the Gamma 3 and Intertan, respectively. The femur radius of curvature measured 1.2 m ± 0.3 (95% CI [1.1–1.2]), which is smaller than the smallest nail radius of 1.5 m.

Table 3 illustrates the subgroup analysis performed for the sex and femur side. The analysis did not show a significant difference between males or females and left or right except for femur neck axis length, which was longer for males than females (*p* < 0.001). The measures of proportion or angle did not show any significant difference between males and females. Table 4 compares measures with the results from other geographical regions. Relative to NSA and Curvature, the measured anteversion mean varied most among the geographic regions of Asia, North America, and Europe with 18.6 ± 7.2, 8.8 ± 9.7, and 10.4 ± 6.7 degrees, respectively.

**Table 3:** Difference between measurements for male/female and left/right subgroups.

	Male (*n* = 56)	Female (*n* = 44)	*p*-value	Left (*n* = 49)	Right (*n* = 51)	*p*-value
	Mean ± *SD*	Mean ± *SD*		Mean ± *SD*	Mean ± *SD*	
Femur neck-shaft axis offset (mm)	6.2 ± 1.6	5.9 ± 1.8	0.392	6.2 ± 1.5	5.9 ± 1.8	0.377
GT to sLSNI (mm)	41.0 ± 5.9	39.5 ± 3.8	0.127	40.4 ± 3.9	40.3 ± 6.1	0.905
Femur neck axis length (mm)	98.0 ± 6.6	89.1 ± 5.0	<0.001	92.9 ± 6.5	95.2 ± 8.0	0.118
NSA of proximal femur (°)	126.2 ± 5.5	126.5 ± 5.4	0.751	126.5 ± 4.9	126.2 ± 6.0	0.769
NSA to apex of femur bow (°)	126.4 ± 5.5	126.7 ± 5.7	0.838	126.6 ± 5.1	126.5 ± 6.0	0.878
NSA to DICN (°)	128.2 ± 5.6	128.4 ± 5.7	0.798	128.3 ± 5.2	128.3 ± 6.0	0.982
Radius of curvature (m)	1.2 ± 0.3	1.1 ± 0.3	0.434	1.2 ± 0.3	1.2 ± 0.3	0.947
Femur anteversion (°)	19.1 ± 9.6	18.4 ± 8.8	0.692	18.11 ± 8.4	19.4 ± 10.0	0.480

Abbreviations: *n*, number; *SD*, standard deviation; mm, millimeter; NSA, neck-shaft angle; DICN, distal intercondylar notch; m, meter; GT, greater trochanter tip; sLSNI, shaft lag screw nail interface.

**Table 4:** Difference between measurements for geographical regions.

	Neck shaft angle (°)	Anteversion (°)	Femur radius of curvature (m)
South Africa (current study)	126.3 ± 5.4	18.8 ± 9.2	1.2 ± 0.3
Asia	128.8 [31]	18.6 ± 7.2 [27]	0.9 ± 0.2 [32]
North American	124.6 [31]	8.8 ± 9.7 [28]	1.1 ± 0.3 [33]
Europe	130.8 ± 6.5 [26]	10.4 ± 6.7 [34]	

Data is described as means ± standard deviations. References are indicated in square brackets.

Abbreviations: m, meter.

## Discussion

Proximal femur fractures frequently require CMNs for fixation; however, geometrical discrepancies between anatomy and implants often curtail successful surgery [12, 13]. This is especially problematic when intraoperative nail options cannot compensate for aspects such as femoral curvature or femur neck anteversion [8]. This study identified that the femur neck and shaft axis offset (FNSAO), the radius of curvature, and femoral anteversion did not overlap the ranges offered by the sampled CMNs. Clinically significant differences between sex could not be identified.

Although this study contributes novel information about the local geographical population, some limitations should be considered in interpreting the findings. This investigation has produced femur geometry measurements with multiplanar reconstruction techniques to limit CT positioning error and ultimately identify anatomy-implant parameter discrepancy [14]. However, the measurement methodology was technical and time-consuming and thus limited multiple authors to limit interobserver reliability [14–16]. Inconsistency of landmark definitions in the reviewed literature complicates the ability to compare our results with pre-existing normal values [17]. In addition, the measures were collected from the Western Cape region of South Africa and may therefore not necessarily extrapolate to other geographic areas.

**Figure 1:**
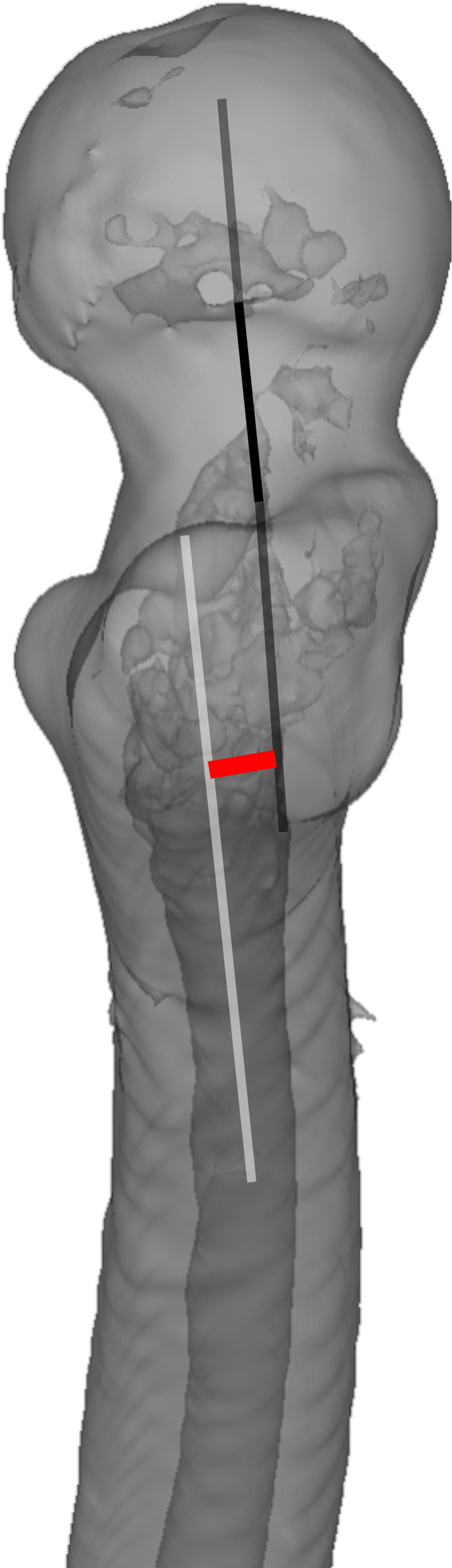
This image shows a lateral view of the proximal femur and illustrate an example of the femur neck-shaft axis offset. Black Line from the femur head: Illustrates the femur neck axis; White line extending into the femur medullary canal: Illustrates the proximal femur axis; Red short line: Illustrate the femur neck-shaft axis offset.

**Figure 2:**
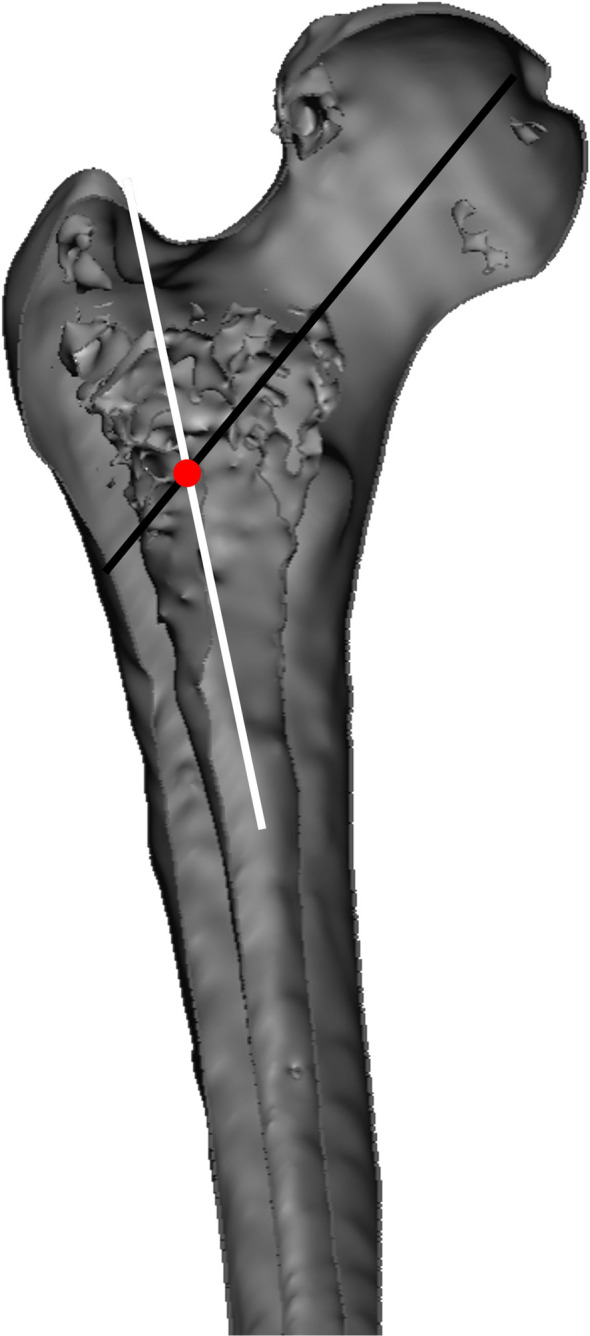
Image shows a bisected anterior view of the proximal femur and depicts the femur neck-shaft axis illustrated as intersecting lines. Black line from the femur head: Illustrates the femur neck axis; White line exiting the greater trochanter: Illustrates the proximal femur axis; Red circle: Illustrate the femur neck-shaft axis offset.

The FNSAO occurring in the sagittal plane was an average of 6.1 mm (*SD* = 1.7). The pseudo-interaction was 40.0 mm (*SD* = 5.1) distal to the tip of the greater trochanter (GT). While the FNSAO to GT measurement was relatively equal to the CMN proximal nail end distances (Table 2), previous studies have listed the proximal nail prominence as a concern for morbidity [10]. The FNSAO, according to our knowledge, has not been quantified previously (Figures 1 and 2). The interaction between the femur neck and shaft axis has been repeatedly studied, but results are confounded due to a myriad of definitions, predominantly due to the complex 3D structure of the femur [17]. Kingsley and Olmsted in 1948 disregarded the center of the femoral head during anteversion measurement due to the concern of femoral head-neck offset [18]. Hoiseth and Fonstelien in 1989 used the center of the femur head but suggested a difference in anteversion dependent on the use of the head-neck or head-shaft axis [19]. The head-shaft axis is not historically the accepted definition of the femur neck, but CMNs rely on a lag screw positioned according to the head-shaft axis [17, 19]. However, lag screw placement in the center of the neck is desired to avoid neck cortex abutment with the screw and, by extension, fracture displacement. CMN design does not appear to accommodate the FNSAO, thus likely complicating sagittal nail placement. To aid correct placement in the sagittal plane, recommendations have been offered for the sagittal entry point [5, 6, 20]:

- At the junction of the anterior ⅓ and posterior ⅔ of the greater trochanter.
- At the tip of the greater trochanter [21].
- In line with the femur medullary canal [5, 22].

**Figure 3:**
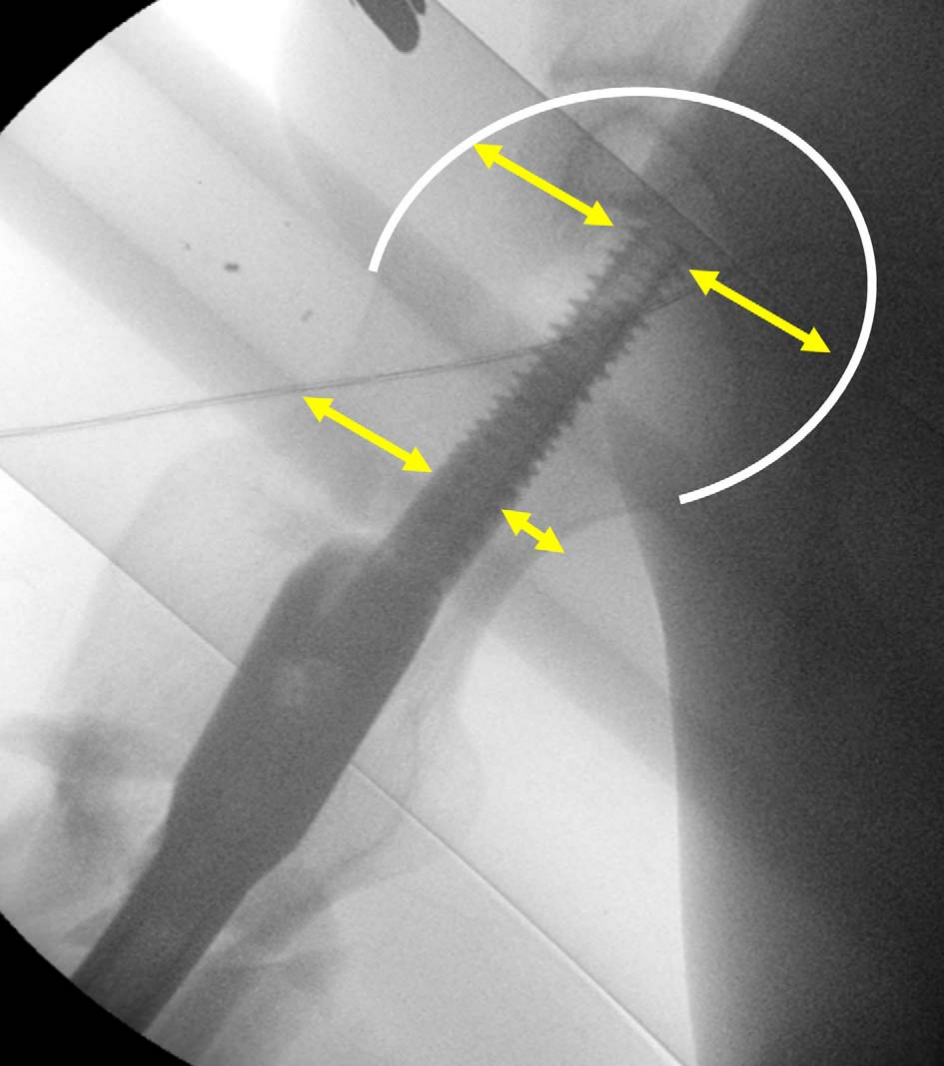
Image is a lateral femur X-ray with a cephalomedullary nail situated after an entry point in line with the femur neck axis resulting in an eccentric neck position. The yellow arrows illustrate disproportionate neck width on either side of the lag screw and equidistance in the femur head.

**Figure 4:**
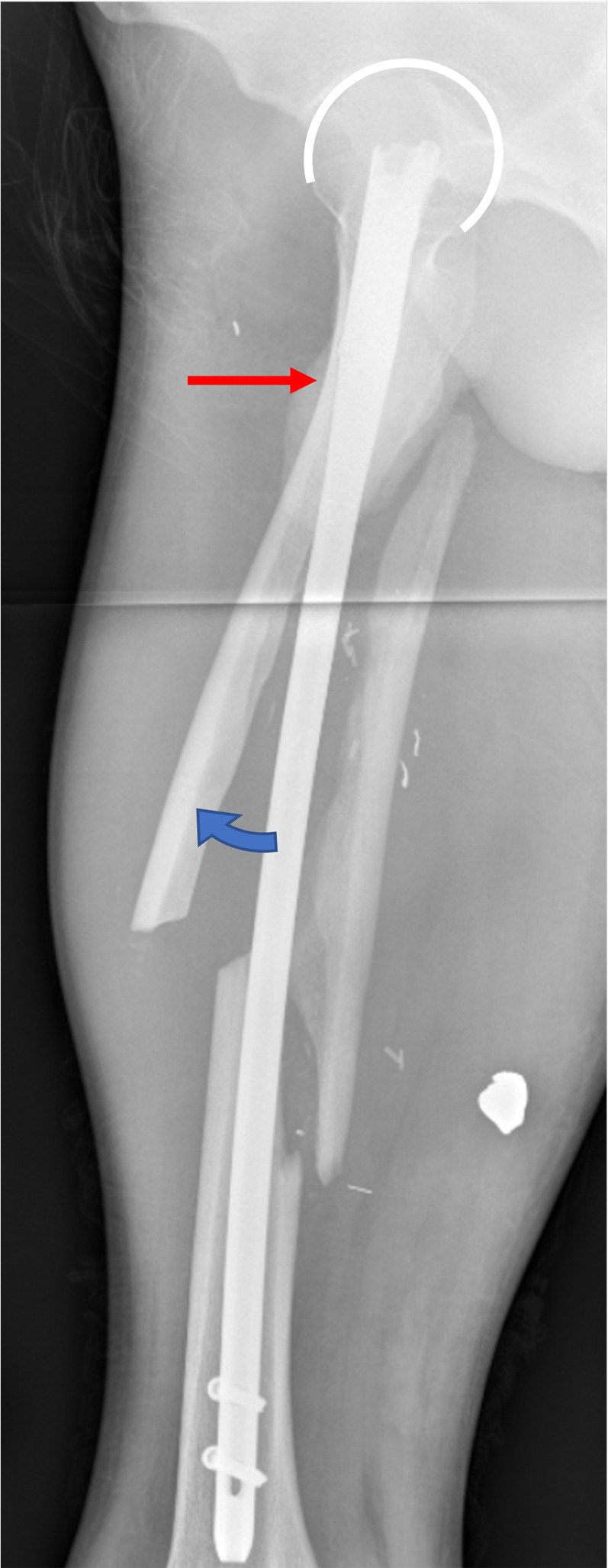
Image is a lateral femur X-ray with a cephalomedullary nail resulting in fracture displacement irrespective of implant placement aligned with the femur neck. The red arrow illustrates proximal anterior femur abutment, and the bottom blue arrow illustrates the ensuing procurvatum.

An entry point in line with the proximal femur axis neglects the anterior neck axis offset, while an entry point on the anterior ⅓ and posterior ⅔ of the greater trochanter is more appropriate for the neck axis but may lead to nail impingement on the anterior femur shaft cortex (Figures 3 and 4) [23]. Stryker’s Gamma 3 (Kalamazoo, Michigan, USA) operative guide suggests the anterior ⅓ posterior ⅔ entry point in elderly patients, likely due to increased medullary canal width [6, 24]. This investigation observed no relationship between the FNSAO and age nor any other measured parameters and may thus be considered a persistent feature in proximal femur anatomy, but the FNSAO impact may be lessened with a wider femur canal. The axis offset may significantly complicate CMN lag screw placement in fracture patterns unable to conform or compensate for the FNSAO, such as in the case of subtrochanteric fractures of young patients.

NSA and anteversion are measures of interaction between the femur neck and shaft axis. This investigation reported NSA corrected for anteversion (true NSA) with three femur axis definitions to compare pre-existing literature. The NSA in this study is unique while based on the two non-intersecting axes previously discussed. To our knowledge, no previous research compared NSA measurements of the proximal, half, and complete femur definitions. The subtle differences were interesting to note, but regardless of definition, were within our institution and published literature parameters [25, 26]. Although larger cohorts have been studied, the measurement methodology did not necessarily compensate for anteversion or include the entire femur [25, 26].

The femur neck anteversion within this study, while larger than previous studies, falls within normal acceptance [17]. Lee et al. [27] described a gold standard measurement method, like the methods employed during this work, and found an average of 18.5° (*SD* = 7.2). Koerner et al. [28] used a single plane for analysis and found a smaller anteversion of 8.8° (*SD* = 9.7). This was likely the reason for the discrepancy in results; however, the multiplanar reconstructed method, as in this study, has been proven to be more accurate [14]. The anteversion measured 18.8° (*SD* = 9.2), which was larger than that offered by the sampled CMNs. The anteversion anatomy-implant mismatch may be compensated by rotating the nail to match the excess native anteversion. However, the nail has a curvature which may affect distal implant placement when the surgeon rotates the nail. The less than required CMN anteversion will likely entail medialization of the distal nail end and be more pronounced in CMNs with a small radius of curvature. This concept requires further investigation.

Femur curvature is not limited to the sagittal plane; thus, using the intramedullary canal point of maximal deviation from the anatomical axis allowed the measurement of femur curvature regardless of the plane of maximal deviation [29]. The intramedullary femur radius of curvature measure was smaller than the offering by the Gamma 3 and Intertan CMN [5, 6]. The theme of curvature mismatch and distal femur anterior cortex abutment has been raised repeatedly in literature [8, 30]. Schmutz et al. attempted to compare femur radius of curvature of various ethnicities and raised the concern that the average femur radius of curvature is smaller than the 1.5 m CMN radius offering [30]. While this study cannot confirm the ethnicity of its sample group, the cohort was from an African population not previously included in femur canal curvature studies. The results echo Schmutz’s concern for anatomy-implant mismatch and should alert a surgeon to identify curvature mismatch preoperatively and use CMNs with a smaller radius of curvature or a short CMN [30]. Parameter mismatch may be due to intentional design philosophy, and for instance, the decreased CMN radius of curvature design may be a response to limit the effect of nail rotation by the surgeon to match native anteversion.

The selected cohort represents a sample from a South African population that is not well represented in traditional implant design [30]. Table 4 compares the results of NSA, Anteversion, and Femur Radius of curvature to other geographical regions. Previous studies have attempted to ascribe anatomy-implant mismatches to demographic variance; however, several mismatches have been shown to span geographical regions [8, 10, 30]. NSA is a well-documented and established parameter allowing comparison across geographical regions [25, 26]. The NSA measurement of this South African cohort was in keeping with accepted parameters [26]. FNSAO, regardless of being a novel measurement, should thus be a concern for other geographical regions. Differences between sex were observed in length parameters; however, measures based on angles or proportions showed no difference. Age has a proven correlation with femur geometry [17, 25], but the present study did not identify a relationship. This was likely due to the low average age of the cohort, which was younger than similar studies [26, 29]. However, the average age was in keeping with the profile of patients presenting to a high-volume trauma unit [2].

## Conclusion

This study reports femur geometry dimensions based on positionally corrected computed tomography images. Device parameters compared in this study that did not overlap the anatomical ranges in our study include the neck-shaft axis offset, radius of curvature, and femoral anteversion. We have highlighted the novel quantified measure of anterior translation of the femur neck that needs consideration during implant placement in the sagittal plane. Clinically relevant demographic differences could not be identified. We suggest considering these measurements for future implant design and surgical techniques to limit anatomy-implant mismatches.

## Conflict of interest

Each author certifies that they have no commercial associations (e.g., consultancies, stock ownership, equity interest, patent/licensing arrangements, etc.) that might pose a conflict of interest in connection with the submitted article.

## Funding

This research received no specific grant from any funding agency in the public, commercial, or not-for-profit sectors.

## Ethical approval

Ethical approval for this study was obtained prior to commencement of data collection, with the approval number S20/02/049 from the Health Research Ethics Committee (REC-203208-010). The Health Research Ethics Committee complies with the SA National Health Act No. 61 of 2003 as it pertains to health research. The HREC abides by the ethical norms and principles for research, established by the World Medical Association (2013).

## Informed consent

The requirement for informed consent was waived due to the retrospective nature of the study.

## Authors contributions

AC: Conceptualization, Writing Original Draft, Investigation; MB: Methodology, Statistics, Reviewing, Supervision; NF: Reviewing and Editing, Supervision; JJ: Reviewing and Editing, Investigation, Supervision.

## Supplemental online material

The Supplemental Online Material of this article is available at https://www.sicot-j.org/10.1051/sicotj/2022004/olm. *Supplemental Online Material 1*: Text outlining the steps followed to obtain the measures. doc *Supplemental Online Material 2*: Table Listing Correlations Between Various Measures and the Femur Neck-Shaft Axis Offset
