# Supplementary material for: Proximal femur anatomy-implant geometry discrepancies
Source: SICOT J. 2022 Mar 4;8:5. doi: 10.1051/sicotj/2022004 (PMC8895924; doi:10.1051/sicotj/2022004)
Supplement: Supplementary file 1 — Supplemental Online Material 1: Text outlining the steps followed to obtain the measures. doc [file sicotj-8-5-s1.pdf]

Supplemental Online Material 1

The images were aligned to the established anatomical axis, allowing identification of the most proximal point of the trochanter and defined as the greater trochanter tip (GT). While maintaining anatomical axis alignment, the coronal view was positioned to identify the most posterior slices with pixels from both posterior condyle pixels still visible to identify the medial and lateral posterior condyle (MPC & LCP), thereby establishing the posterior condylar axis. The lateral point on the distal femur was defined by the lateral epicondyle (LE), while the sulcus behind the medial epicondyle on the axial view was defined as the surgical medial epicondyle (sME). A line between LE and sME established the surgical trans-epicondylar axis (sTEA).
The center of a best-fitted sphere based on sagittal, coronal, and axial views of the femoral head center (COFH) defined the proximal mechanical axis point. The mechanically aligned planes were focused on the COFH and, after that, realigned on axial and coronal views to bisect the neck at its narrowest point and thereby define the femoral neck axis (FNA). The FNA was used to plot various points. The most medial point on the femur neck axis was defined as the tip of the femur head (TOFH). The lateral point on the same axis was defined as the Lateral Femur Neck Axis Length Point (LFNAL).

We used the sagittal coronal and axial plane images to identify a medullary point of maximal deviation from the anatomical axis defined as the femur bow apex. Next, we used the axial views of the femur medulla to accurately place the three mid-medullary points required to calculate the radius. Finally, a formula was used to produce the radius of a circular Segment (R), by using the distance between MOCLT and DICN (c), and the most significant distance from the femur anatomical axis to the femur bow apex (h):

$$R = \frac{h}{2} + \frac{c^2}{8h}$$

The proximal femur anatomical axis (PFAA) was established with a line between MOCLT and a point placed 50mm distally in the central medullary canal. Next, the shortest distance between the FNA and PFAA was defined on the PFAA as the shaft and lag screw interface point (sLSNI), and the corresponding point on the neck axis was defined as the (nLSNI). Next, the distance

between the nLSNI and sLSNI was measured to establish the femur-neck shaft axis offset distance (FNSAO).

Femur NSA was measured with three definitions. The femur neck axis remained constant while the femur shaft axis was defined between the MOCLT and a distal point 50mm distal to the LT, a distal point at the apex of the femur bow, and a distal point at the DICN. The three different angles were termed NSA of the proximal femur, NSA to the apex of the femur bow, and NSA to the DICN, respectively.

The surgical trans-epicondylar angle was measured between the trans-epicondylar axis and the posterior condylar axis (a line connecting the MPC and LPC). Next, the femoral anteversion and surgical trans-epicondylar angles were measured in two-dimensional views while the plane was aligned with the femur anatomical axis. Finally, the axial view was sequentially focused on the previously identified COFH, LFNAL, LFNAL, MPC, LPC, sME, and LE. First, the points were placed on the same plane as the MPC point; then, the femoral anteversion and sTEA angles were measured.
