# Supplementary material for: Proximal femur anatomy-implant geometry discrepancies
Source: SICOT J. 2022 Mar 4;8:5. doi: 10.1051/sicotj/2022004 (PMC8895924; doi:10.1051/sicotj/2022004)
Supplement: Supplementary file 2 — Supplemental Online Material 2: Table Listing Correlations Between Various Measures and the Femur Neck-Shaft Axis Offset [file sicotj-8-5-s2.pdf]

Supplemental Online Material 2. Table Listing Correlations Between Various Measures and the Femur Neck-Shaft Axis Offset

Measurement Description	Pearson's r / Spearman rank rho	p-value
GT to sLSNI (mm)	-0.109	0.279
Femur Neck Axis Length (mm)	0.054	0.591
NSA of proximal femur (degrees)	-0.113	0.265
NSA to apex of femur bow (degrees)	-0.106	0.296
NSA to DICN (degrees)	-0.118	0.242
Radius of Curvature (m)	-0.027	0.791
Femoral Anteversion (degrees)	-0.107	0.291

Abbreviations: SD, Standard Deviation; 95% CI, 95 Percent Confidence Interval; GT, Greater Trochanter Tip; MOCLT, middle of canal at lesser trochanter; sLSNI, shaft lag screw nail interface; NSA, Neck Shaft Angle; DICN, Distal intercondylar Notch; °, Degrees; yrs, years; mm, millimeter; m, meter; N/A, not applicable.

[a] Non-parametric data reported as median (interquartile range).
